# P56 Involvement of young people in adolescent and young adult rheumatology services and research

**DOI:** 10.1093/rap/rkac067.056

**Published:** 2022-09-28

**Authors:** Beth Dillon, Suruthi Gnanenthiran, Megan Stubbs, Sarah Yorke, Lunt E Laura, McDonagh E Janet

**Affiliations:** Your Rheum, a national advisory group of the Barbara Ansell National Network for Adolescent Rheumatology, Manchester, United Kingdom; Your Rheum, a national advisory group of the Barbara Ansell National Network for Adolescent Rheumatology, Manchester, United Kingdom; Your Rheum, a national advisory group of the Barbara Ansell National Network for Adolescent Rheumatology, Manchester, United Kingdom; Versus Arthritis, London, United Kingdom; Centre for Epidemiology Versus Arthritis, Centre for Musculoskeletal Research, Manchester Academic Health Science Centre, University of Manchester, Manchester, United Kingdom; NIHR Manchester Biomedical Research Centre, Manchester University NHS Foundation Trust, Manchester, United Kingdom; Centre for Epidemiology Versus Arthritis, Centre for Musculoskeletal Research, Manchester Academic Health Science Centre, University of Manchester, Manchester, United Kingdom; NIHR Manchester Biomedical Research Centre, Manchester University NHS Foundation Trust, Manchester, United Kingdom

## Abstract

**Introduction/Background:**

The Barbara Ansell National Network for Adolescent Rheumatology (BANNAR) was established in 2012. Survey results from within the paediatric and adolescent rheumatology community at this time, highlighted the lack of young people’s involvement in rheumatology research, beyond the role of study participant. Following research to determine the young person’s perspective of research and how they wanted to be involved, Your Rheum, a national young person’s advisory group, for 11-24 year olds diagnosed with a rheumatic condition, was formed in 2016.

**Description/Method:**

Aim: to assess the current involvement of young people in rheumatology research and service delivery. A questionnaire was created using Microsoft Office online forms and emailed to all BANNAR members (n = 105). Topics included demographics, youth involvement panels and Your Rheum.

**Discussion/Results:**

23 responses were received (21% response rate), representing 18 rheumatology centres across the UK including 15 tertiary paediatric and adolescent rheumatology centres. The majority of respondents were Consultants (n = 18). Over half of respondents (n = 16) work in centres involved in rheumatology research.

15 centres reported having a hospital-wide youth forum/advisory panel, which they promote amongst young people within their services. However, nearly all respondents reported no specific youth advisory panel for rheumatology services. Young people are actively involved in a number of areas within clinical rheumatology services (table 1), however patient satisfaction surveys were the most commonly reported. Moreover, young people are involved in aspects of rheumatology research (table 1), although this was primarily as research participants.

Over half of respondents (n = 14) have not worked with Your Rheum. Most recruit young people to get involved in the group (n = 16), however, only three respondents said they do this routinely. Common barriers to promoting Your Rheum were: lack of time in consultations; forgetting; lack of interest from the young person(s); no designated member in clinic to discuss Your Rheum with young people.

**Key learning points/Conclusion:**

There remains a need to support youth involvement strategies at a local level as well as nationally via Your Rheum. The recent introduction of a Your Rheum animation (https://bit.ly/yourRHEUM) will hopefully support future recruitment.

**P56 Table 1:** 

What aspects of your CLINICAL rheumatology service do young people actively participate in currently?	Count
Patient satisfaction surveys	10
Service design and development	8
Recruitment and interviewing of new clinical staff	5
Training of health professionals	2
Undergraduate education	1
Reviewing local service provision against criteria other than the You're welcome criteria	1
Other, (development of app)	1
What aspects of your rheumatology RESEARCH do young people actively participate in?	Count
Research participants in relevant projects	9
Developing protocols	5
Membership of an advisory board	5
Design of interventions	4
Setting the research agenda	3
Recruitment and interviewing of new research staff who will have direct contact with young people	3
Dissemination of results	3
Co-researchers	3

